# Why Can Only 24% Solve Bayesian Reasoning Problems in Natural Frequencies: Frequency Phobia in Spite of Probability Blindness

**DOI:** 10.3389/fpsyg.2018.01833

**Published:** 2018-10-12

**Authors:** Patrick Weber, Karin Binder, Stefan Krauss

**Affiliations:** Mathematics Education, Faculty of Mathematics, University of Regensburg, Regensburg, Germany

**Keywords:** Bayesian reasoning, natural frequencies, probabilities, einstellung, tree diagram

## Abstract

For more than 20 years, research has proven the beneficial effect of natural frequencies when it comes to solving Bayesian reasoning tasks (Gigerenzer and Hoffrage, 1995). In a recent meta-analysis, McDowell and Jacobs (2017) showed that presenting a task in natural frequency format increases performance rates to 24% compared to only 4% when the same task is presented in probability format. Nevertheless, on average three quarters of participants in their meta-analysis failed to obtain the correct solution for such a task in frequency format. In this paper, we present an empirical study on what participants typically do wrong when confronted with natural frequencies. We found that many of them did not actually *use* natural frequencies for their calculations, but translated them back into complicated probabilities instead. This switch from the intuitive *presentation format* to a less intuitive *calculation format* will be discussed within the framework of psychological theories (e.g., the Einstellung effect).

## Introduction

Many professionals, such as medical doctors and judges in court, are expected to make momentous decisions based on statistical information. Often, Bayesian inferences are required, for example when a radiologist has to judge and communicate the statistical meaning of a positive mammography screening. Many empirical studies have documented faulty inferences and even cognitive illusions among professionals of various disciplines (Hoffrage et al., 2000; Operskalski and Barbey, 2016). In the medical context, the consequences are particularly severe because many patients are mistakenly found diseased, which can entirely change their lives (Brewer et al., 2007; Gigerenzer et al., 2007; Salz et al., 2010; Wegwarth and Gigerenzer, 2013). Similarly, insufficient knowledge of statistics in general and incorrect Bayesian reasoning in particular can result in false convictions or acquittals made by juries in court, for example when they have to evaluate evidence based on a fragmentary DNA sample. These faults bear the risk of destroying innocent people's lives, too, as happened, for instance, in the famous case of Sally Clark (Schneps and Colmez, 2013; Barker, 2017).

Typically, the statistical information that the aforementioned professionals are confronted with is provided in probability format, that is, fractions or percentages describing the probability of a single event, for example the prevalence of breast cancer in the population. Generally, in situations where Bayesian inferences are necessary, three pieces of statistical information are given: the base rate (or a priori probability), sensitivity, and false alarm rate. Consider, for instance, the heroin addiction problem (adapted from Gigerenzer and Hoffrage, 1995):

Heroin addiction problem (probability format):

The probability of being addicted to heroin is 0.01% for a person randomly picked from a population *(base rate)*. If a randomly picked person from this population is addicted to heroin, the probability is 100% that he or she will have fresh needle pricks *(sensitivity)*. If a randomly picked person from this population is not addicted to heroin, the probability is 0.19% that he or she will still have fresh needle pricks *(false alarm rate)*. What is the probability that a randomly picked person from this population who has fresh needle pricks is addicted to heroin (*posterior probability*)?

With the help of Bayes' theorem, the corresponding posterior probability P(H|N), with H denoting “person is addicted to heroin” and N denoting “person has fresh needle pricks,” can be calculated.

(1) $$\begin{array}{l} P\left(H|N\right)\;=\;\frac{P\left(N|H\right)\;\cdot \;P\left(H\right)}{P\left(N|H\right)\;\cdot \;P\left(H\right)\;+\;P\left(N|\neg H\right)\;\cdot \;P\left(\neg H\right)} \end{array}$$

$$\begin{array}{l} \;=\;\frac{100\%\;\cdot \;0.01\%}{100\%\;\cdot \;0.01\%\;+\;0.19\%\;\cdot \;99.99\%}\;\approx \;5\%\; \end{array}$$

Given the probabilistic information (the low base rate, high sensitivity, and low false alarm rate), the result of only 5% seems astonishingly low to most people—professionals and laypeople alike. In fact, only very few—on average as few as 4% of the participants included in a comprehensive meta-analysis (McDowell and Jacobs, 2017)—are able to draw the correct inferences necessary to come to the right conclusion in such Bayesian tasks. The vast majority of people have difficulties, which can result in severe misjudgments.

The reasons for this poor performance in Bayesian reasoning are widely discussed. One explanation is the neglect of the base rate, which can be very low in many Bayesian situations (Tversky and Kahneman, 1974; Bar-Hillel, 1983). This leads to much greater estimates for the posterior probability, which is consistent with most people's intuition. Further reasons for the poor performance include participants neglecting the false alarm rate P(N|H) or confusing the false alarm rate with the posterior probability P(H|N) (Gigerenzer and Hoffrage, 1995) as well as participants overweighing the sensitivity (e.g., McCloy et al., 2007).

In order to prevent dangerous misjudgments due to faulty Bayesian inferences, the concept of *natural frequencies* has proven to be a powerful instrument (e.g., Gigerenzer and Hoffrage, 1995; Siegrist and Keller, 2011). Natural frequencies can be obtained by *natural sampling* (Kleiter, 1994) or, alternatively, by translating probabilities (e.g., “80%”) into expressions consisting of two absolute frequencies (e.g., “80 out of 100”; for a discussion on the equivalence of natural frequencies and probabilities, see section Present Approach). Consider once again the heroin addiction example, this time, however, in natural frequency format:

10 out of 100,000 people from a given population are addicted to heroin. 10 out of 10 people who are addicted to heroin will have fresh needle pricks. 190 out of 99,990 people who are not addicted to heroin will nevertheless have fresh needle pricks. How many of the people from this population who have fresh needle pricks are addicted to heroin?

With the help of this format, significantly more people find the correct answer to the problem, which is 10 out of (10 + 190). As a consequence, performance rates in the frequency format typically increase to about 24% (McDowell and Jacobs, 2017). Errors due to base rate neglect as mentioned above occur less often with natural frequencies, since the base rate need not be attended to in the frequency version because it is already included in the information on the sensitivity and false alarm rate. Thus, Bayes' modified theorem containing natural frequencies yields the correct answer of “10 out of 200” in the heroin addiction problem based on a simpler computation:

(2) $$P\left(H|N\right)=\;\frac{\#(N\cap H)}{\#(N)}=\frac{10}{10+190}=5\%$$

More than 20 years of research have confirmed the benefit that comes with the concept of natural frequencies in Bayesian reasoning situations. Laypeople, students, professionals across various domains (e.g., medicine, law, and management), and even children perform significantly better when working on a Bayesian reasoning task that is presented in natural frequencies instead of probabilities (e.g., Wassner, 2004; Zhu and Gigerenzer, 2006; Hoffrage et al., 2015; Binder et al., 2018).

Additionally, various other factors are known to have an impact on performance in Bayesian reasoning tasks. Visualizations, for example tree diagrams (e.g., Yamagishi, 2003; Binder et al., 2018), unit squares (e.g., Böcherer-Linder and Eichler, 2017; Pfannkuch and Budgett, 2017), icon arrays (e.g., Brase, 2009, 2014) or roulette wheel diagrams (e.g., Yamagishi, 2003; Brase, 2014), have been shown to improve accuracies in Bayesian situations (for an exception, see, e.g., Micallef et al., 2012). An overview and categorization of visualizations that were used to boost performance in Bayesian situations is provided by Khan et al. (2015). Furthermore, individual differences of participants, particularly cognitive abilities such as numeracy, graphicacy, and spatial abilities, certainly have an impact on performance rates (e.g., Chapman and Liu, 2009; Brown et al., 2011; Micallef et al., 2012; Peters, 2012; Ottley et al., 2016). In addition, the specific numerical values for population size, base rate, sensitivity, and false alarm rate can influence accuracies (Schapira et al., 2001). Cognitive biases and judgment errors associated with different numerical information are, for example, size effect and distance effect (Moyer and Landauer, 1967). Finally, details of the representation and framing of the problem text can affect performance in Bayesian reasoning situations (Obrecht et al., 2012). Ottley et al. (2016), for example, were able to show that specific problem formulations (e.g., providing *all* numerical information in context of the task, that is, not only base rate, sensitivity, and false alarm rate but also the probability or frequency of their respective complement) influence accuracies significantly.

However, instead of contributing to the abundance of empirical studies replicating and discussing the beneficial effect of natural frequencies or other factors (e.g., Hoffrage et al., 2002; Pighin et al., 2016; McDowell et al., 2018), in this article we will focus on the other side of the coin, that is, on the 76% of participants in these studies (on average in McDowell and Jacobs, 2017) who failed to solve Bayesian reasoning tasks with natural frequencies. Why can still on average only a quarter of participants solve the problem correctly, although the task is presented in the beneficial natural frequency format? Many psychological theories explain, discuss, and specify in detail if and why natural frequencies facilitate Bayesian inferences (e.g., the nested sets-hypothesis or the ecological rationality framework, see Gigerenzer and Hoffrage, 1999; Lewis and Keren, 1999; Mellers and McGraw, 1999; Girotto and Gonzalez, 2001, 2002; Hoffrage et al., 2002; Sloman et al., 2003; Barbey and Sloman, 2007; Pighin et al., 2016; McDowell et al., 2018) and how additional tools, such as visualizations, further increase their beneficial effect (e.g., Yamagishi, 2003; Brase, 2009, 2014; Spiegelhalter et al., 2011; Micallef et al., 2012; Garcia-Retamero and Hoffrage, 2013; Micallef, 2013; Ottley et al., 2016; Böcherer-Linder and Eichler, 2017). However, a satisfying answer to the question why only 24% of participants solve Bayesian reasoning problems in natural frequency format correctly has not yet been found.

## Present approach

In order to explain why only 24% of participants draw correct Bayesian inferences when confronted with natural frequencies, in the present article we take one step back and switch our focus from *performance rates* to *cognitive processes*. In this respect, some important questions have not been addressed in detail so far: When given a Bayesian reasoning problem in frequency format, how do participants who fail to provide the correct answer approach the task? Where exactly do their calculations fail and why?

In order to gain a first impression of what participants might do when confronted with a task in natural frequency format, we checked the questionnaires from our previous studies on Bayesian reasoning and natural frequencies (e.g., Krauss et al., 1999; Binder et al., 2015). Interestingly, we revealed some instances where participants had not applied the given natural frequencies but had translated them back into probabilities. In order to explore this phenomenon in depth, we had a closer look on what students usually learn about Bayesian reasoning problems in their high school statistics classes.

Over the past two decades, statistics education has become an important column in German high school curricula. Here, just like in other countries, systematic calculation with probabilities has been in the center of teaching efforts. Alternative formats, such as natural frequencies, have despite the great amount of empirical research underpinning their benefits only played a minor role (cf. the American GAISE recommendations; Franklin et al., 2007). Even though there are some very recent efforts to implement the frequency concept in German curricula, for example in the new Bavarian high school curriculum for grade 10 (ISB, 2016), there still seems to be a tendency that this format is not accepted as equally mathematically valid as probabilities. This is supported by our impression from trainings for mathematics teachers that the concept of natural frequencies is not even familiar to most teachers. Furthermore, many schoolbooks tend to solve statistical tasks (not only Bayesian ones) with probability calculations, even when the task is presented in absolute frequencies (e.g., Freytag et al., 2008; Rach, 2018). Another observation we made based on a review of typical Bavarian school textbooks (Eisentraut et al., 2008; Freytag et al., 2008; Schmid et al., 2008) and workbooks (Sendner and Ruf-Oesterreicher, 2011; Reimann and Bichler, 2015) was that the more advanced students become in their high school career, the fewer statistical tasks are solved with natural frequencies by the respective textbooks. In conclusion, high school (and, consequently, university) students are a lot more familiar with probabilities than with natural frequencies due to their general (and sometimes even tertiary) statistical education. This implies that working with probabilities is a well-established strategy when it comes to solving statistical problems.

While in many situations people profit from such an established strategy, in some cases, however, a previously fixed mindset can block simpler ways to approaching a problem (Haager et al., 2014). This phenomenon lies at the center of prominent psychological theories on cognitive rigidity. Consider, for example, the so-called Einstellung or mental set effect (Luchins, 1942). When solving a problem, people often rigidly apply a previously learnt solution strategy while neglecting possibly important information that would allow an easier solution. Such an *Einstellung* or *mental set* can be developed through repeated training, enabling the person to quickly solve problems of the same structure (Schultz and Searleman, 2002; Ellis and Reingold, 2014; Haager et al., 2014). However, the downside of these mental sets is that they can make a person “blind” to simpler solutions or—in the worst case—unable to find a solution at all.

The most famous example for the Einstellung effect is Luchin's water jar experiment (1942; for more recent studies on the Einstellung effect in chess players and with anagram problems see, e.g., Bilalić et al., 2008; Ellis and Reingold, 2014). Participants in Luchin's study had to work out on paper how to obtain a certain volume of water using three empty jars of different sizes for measuring. The first five problems could all be solved by applying a relatively complicated strategy that was shown to the participants in an example problem. For the following five problems, a much simpler solution method was possible. However, the majority of participants kept using the complicated strategy they had previously learnt. Moreover, many of them could not solve the eighth problem at all, for which only the simple solution strategy was appropriate (Luchins, 1942).

Recent research has shown that even experts can be subject to the Einstellung effect (e.g., Bilalić et al., 2008). Thus, mental sets developed over a long period of time can also lead to the blocking of simple solutions (for a detailed discussion of different aspects of cognitive rigidity see Schultz and Searleman, 2002). The probability strategy, which German students deal with during their whole high school career, would be an example for such a mental set that is developed over time. So taken together, these psychological theories and the strong familiarity of students with probabilities hint toward a possible answer to the question what participants might wish to do when they are confronted with a task in frequency format: They might try to represent the situation in the much more familiar probability format in order to be able to use established probabilities for their calculations.

Such an Einstellung toward calculating with probabilities instead of natural frequencies would take away all benefits that come with the frequency concept. Calculating with probabilities in a Bayesian context—even though the task is provided in frequency format—has the consequence that the intuitive natural frequency algorithm [formula (2)] is no longer available, the more complicated probability algorithm [formula (1)] has to be applied, and people are no longer able find the correct solution. Thus, the Einstellung effect might explain why on average three quarters of participants fail with natural frequencies. In the same line, we assume that it is very unlikely that people translate probabilities into natural frequencies when given a task in probability format—despite over 20 years of research on the beneficial effects of natural frequencies.

Here, the question might arise whether the two formats can actually be considered equivalent. In this respect, both mathematical and psychological aspects need to be addressed. First, we will shed light on the respective mathematical frameworks both formats operate in and to what extent these frameworks can be considered equivalent. Second, we will analyze the equivalence of probabilities and natural frequencies from a psychological viewpoint.

Even though the two formats seem to follow different rules, from a mathematical perspective they can be defined analogously. Weber (2016) showed that natural frequencies can be embedded in a theoretical framework that is isomorphic to a probability space, that is, the structure at the basis of probability theory can be constructed in a similar way for natural frequencies. Thus, all fundamental mathematical properties of probabilities, for example closure, commutativity, and associativity of their addition, can theoretically also be assigned to natural frequencies (for details, see Weber, 2016). Therefore, the two concepts can be considered equivalent, implying that natural frequencies are an information format just as mathematically valid as probabilities.

However, regardless of this theoretical equivalence of the two formats, a certain psychological uneasiness about the equivalence of natural frequencies and probabilities still seems to exist. It can be speculated that students who do not know about the mathematical framework of the frequency format might switch from natural frequencies to probabilities not only because they think that a probability algorithm is the only or the easiest way to solve the problem but also due to this subtle feeling of uneasiness, which stems from the assumption that natural frequencies are not a mathematically valid tool for solving Bayesian reasoning tasks. The latter implies that participants—even if they realize that a solution can be derived very easily by using natural frequencies—might think that a mathematically justified argumentation requires reasoning in terms of probabilities. All three assumptions (probabilities are the only, the easiest or the only allowed way) might trigger participants to rely on their Einstellung instead of actively using natural frequencies.

To be clear, we theoretically consider natural frequencies as a superordinate concept for both “expected” and “empirically sampled” frequencies. Expected frequencies constitute frequencies expected in the long run (cf. Hertwig et al., 2004; Spiegelhalter and Gage, 2015; case 2 in Woike et al., 2017) and are often used for problem formulations in natural frequency format. In contrast, empirically sampled frequencies are derived from a natural sampling process (cf. Kleiter, 1994; Fiedler et al., 2000; cases 1 and 3 in Woike et al., 2017; for a discussion of the two sub-concepts of natural frequencies, see also Hertwig et al., 2004; Spiegelhalter and Gage, 2015).

Of course, in the context of possibly switching between the two formats, besides the information format of the task, also the format in which the *question* is asked has to be taken into consideration (for a discussion on other details of textual problem representation, see, e.g., Ottley et al., 2016). It has to be noted that several studies (e.g., Cosmides and Tooby, 1996; Evans et al., 2000; Girotto and Gonzalez, 2001; Sirota et al., 2015) suggest that a question format that does not match the information format of the task reduces the natural frequency facilitation effect (Ayal and Beyth-Marom, 2014; Johnson and Tubau, 2015). However, only few studies directly test such incongruent problem and question formats (McDowell and Jacobs, 2017).

We also do not want to examine incongruent formats (or other factors mentioned above) systematically (e.g., in order to boost performance), but rather aim to implement a question format as neutral as possible that allows for both answer formats simultaneously. Our interest is to observe and analyze a substantial amount of participants for all four possible cases, namely those who stay with the given format (probability or natural frequency) and those who switch to the other format for their calculations, in order to learn from the respective cognitive processes about possible mechanisms underlying the choice of calculation format.

Since in our questionnaires from previous studies (Krauss et al., 1999; Binder et al., 2015), it was not always possible to judge which calculation format a participant applied, we will now explicitly ask participants to write down their solution algorithm in order to capture cognitive policies. Thus, in the present study we enter new research fields by investigating potential preferences in *calculation format*—when a problem introduction and question format as neutral as possible are given—that become visible by the way participants try to solve a given Bayesian task.

Our research questions are:

- Research question 1: Do participants show a general preference of the probability format over natural frequencies that becomes manifest in a strong tendency to

- keep working with probabilities if a task is given in probability format, although a sample population is provided
- even translate a task given in frequency format into probabilities, if the question allows for answers in both formats?

- Research question 2:

- Regardless of the format in which the task is presented, do participants who work on this task actively using natural frequencies make more correct Bayesian inferences than participants who make their computations with probabilities?
- If questions allow for answers in both formats, which factor predicts correct Bayesian inferences better—the format that the task is presented in (*presentation format*) or the format that participants actively use for their calculations (*calculation format*)?

Regarding research question 1, we hypothesized that participants do show a strong preference of probabilities over natural frequencies in both presentation formats. We further assumed that this preference has indeed a detrimental effect on performance in Bayesian reasoning tasks. With regard to research question 2, we therefore hypothesized that actively working with natural frequencies is a stronger predictor for correct inferences than the presentation format of a task.

## Experimental study

To examine these research questions, we conducted an empirical study with a first sample (*N* = 114) in 2016 (see section Participants). In the light of the current debate on the replication crisis (e.g., Open Science Collaboration, 2015), we decided to check the robustness of the results obtained with another sample (*N* = 69) with the same materials and design in 2017/2018. Three participants from the second sample were excluded from the analysis because they indicated that they had already participated in the first sample. Since we detected the same effects for both samples independently, we report the results for the combined sample of *N* = 180 (see section Results).

### Method

Participants in our study had to work on two Bayesian reasoning tasks with different scenarios (heroin addiction problem and car accident problem, adapted from Gigerenzer and Hoffrage, 1995) and different numerical data (for design see Table 1 and for problem wordings see Table 2). These two contexts were chosen since they are not as common as, for example, the famous mammography problem, and thus, the chance of a participant already knowing the task beforehand was small. Moreover, both problems refer to daily-life situations, so the participants were expected to have no difficulties understanding the scenarios. One of the two Bayesian problems was presented in probability format and the other one in natural frequency format. We systematically permuted the order of context as well as information format.

**Table 1:** Design of the implemented problem versions.

		**Context**	
		**Heroin addiction problem**	**Car accident problem**
Presentation format	Probabilities	• Introduction: sample provided • Presentation format of the task: probabilities • Question format: probabilities • Visualization presented or to be constructed	• Introduction: sample provided • Presentation format of the task: probabilities • Question format: probabilities • Visualization presented or to be constructed
	Natural frequencies	• Introduction: sample provided • Presentation format of the task: natural frequencies • Question format: proportions • Visualization presented or to be constructed	• Introduction: sample provided • Presentation format of the task: natural frequencies • Question format: proportions • Visualization presented or to be constructed

**Table 2:** Problem formulations.

	**Heroin addiction problem**		**Car accident problem**	
	**Probability version**	**Natural frequency version**	**Probability version**	**Natural frequency version**
Introduction	Imagine that you randomly meet a person with fresh needle pricks in the street. You are interested in whether this person is addicted to heroin. On the internet, you find the following information for a sample of 100,000 people:		Imagine you see a drunken person getting behind the wheel of his or her car after a party. You are interested in the risk of a car accident caused by this person. On the internet, you find the following information for a sample of 10,000 drivers:	
Statistical information	The probability that one of these people is addicted to heroin is 0.01%. If one of these people is addicted to heroin, the probability is 100% that he or she will have fresh needle pricks. If one of these people is not addicted to heroin, the probability is 0.19% that he or she will nevertheless have fresh needle pricks.	10 out of 100,000 people are addicted to heroin. 10 out of 10 people who are addicted to heroin will have fresh needle pricks. 190 out of 99,990 people who are not addicted to heroin will nevertheless have fresh needle pricks.	The probability that one of these drivers will cause an accident is 1%. If one of these drivers causes an accident, the probability is 55% that he or she is drunk. If one of these drivers does not cause an accident, the probability is 5% that he or she is nevertheless drunk.	100 out of 10,000 drivers cause an accident. 55 out of 100 drivers who cause an accident are drunk. 500 out of 9,900 drivers who do not cause an accident are nevertheless drunk.
Question	What is the probability that one of these people is addicted to heroin, if he or she has fresh needle pricks?	Of the people who have fresh needle pricks, what is the proportion of them addicted to heroin?	What is the probability that one of these drivers causes an accident, if he or she is drunk?	Of the drivers who are drunk, what is the proportion of them causing an accident?
Visual aid	• First task: construct a tree diagram • Second task: consider a presented tree diagram	• First task: construct a tree diagram • Second task: consider a presented tree diagram	• First task: construct a tree diagram • Second task: consider a presented tree diagram	• First task: construct a tree diagram • Second task: consider a presented tree diagram
Prompt	“Please write down your calculations!”			

In typical natural frequency versions, the question reads “How many of the … have/are …?,” often followed by a line “Answer: ____ out of ____.” Note that we are interested in cognitive processes triggered purely by the *presentation format* and not by a provided question or answer format. Thus, in all natural frequency versions, we wanted to implement a question format that allows both for probability and for natural frequency answers. In order to be as neutral as possible, we decided to use questions for *proportions* (see Tables 1, 2), which are a common question format in schoolbooks, too. The question “What is the proportion of people…” can be answered by, for example, “5%” or by “10 out of 200” and thus is settled in between probabilities and natural frequencies.

In the probability versions, formulating a neutral question is rather difficult because a proportion usually refers to a concrete sample. Thus, instead of making the question format as neutral as possible, we decided to provide the participants already in the introduction with a sample population that the probabilities could be referred to (e.g., “On the internet, you find the following information for a sample of 100,000 people”). Thereby, we again allowed for both calculation formats. While in natural frequency versions the option for probability answers lies in the neutral question format, a possible natural frequency answer in probability versions was opened up by providing a concrete sample in the beginning of the task. It is important to note that we did not primarily want to compare performances by *presentation format* (which would just be a replication of many other studies) but by *calculation format*, so a total parallelization of the task versions was neither necessary nor the optimal design for our research questions.

**Figure 1:**
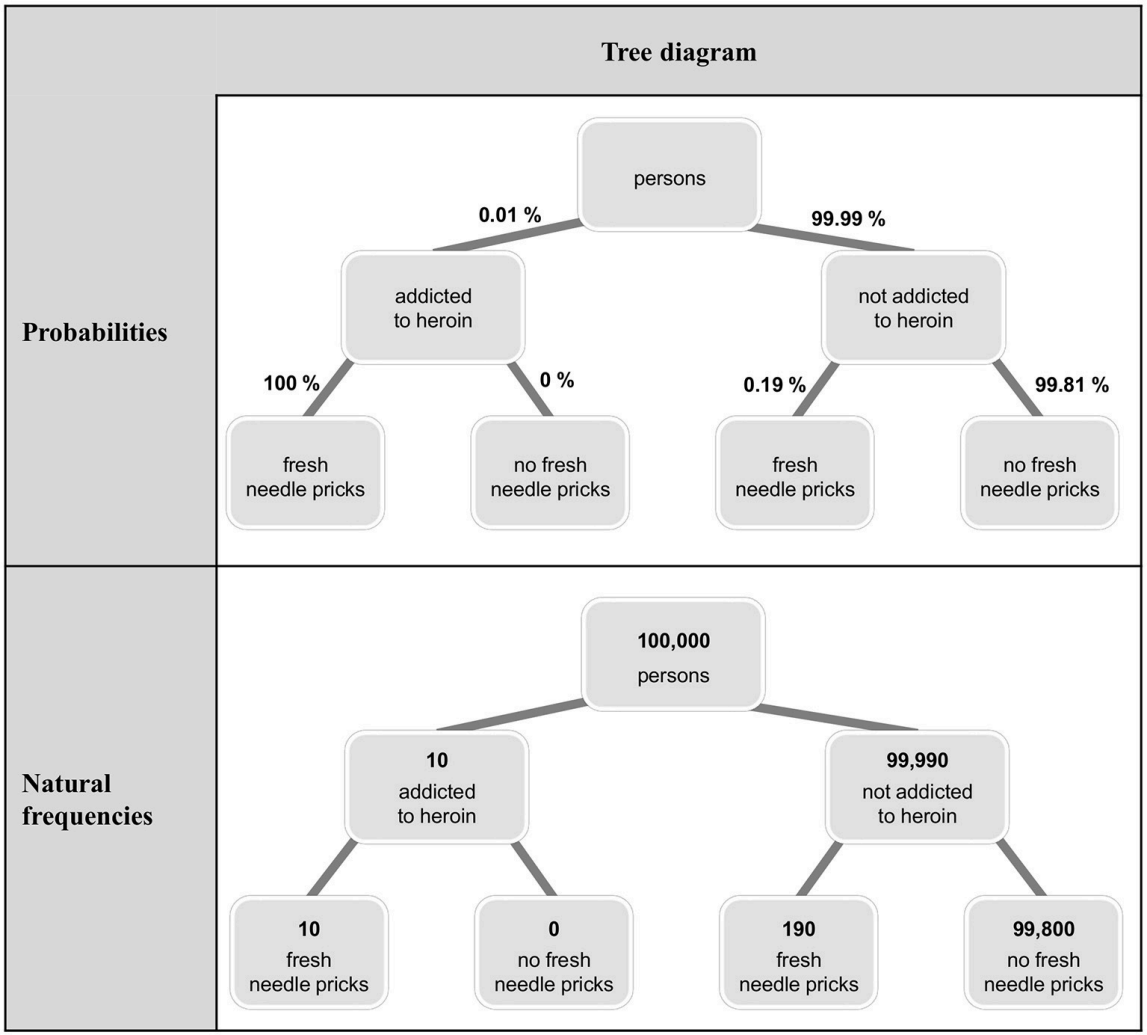
Tree diagrams visualizing the heroin addiction problem equipped with probabilities and natural frequencies.

Because Bayesian reasoning tasks in German schoolbooks are usually presented with tree diagrams (Binder et al., 2015), after the question, we either asked for the construction of a tree diagram (in the first task) or presented a tree diagram (in the second task). The aim here was to present stimuli that are as ecologically valid as possible [with respect to (German) teaching contexts both in school and in university] and that provide the option to switch between the two formats. Both at school and at university level, 2 × 2-tables and tree diagrams are most commonly used for teaching Bayesian reasoning, whereas alternative visualizations (unit squares, icon arrays, etc.) are usually omitted. Since both 2 × 2-tables and tree diagrams allow for switching between the two formats (unlike, e.g., icon arrays) and since tree diagrams but not 2 × 2-tables can be directly equipped with *conditional* probabilities, only tree diagrams remained as visualizations suitable for our study. By using the latter, our hope was to exploratively shed light on whether a tree diagram might influence participants' choice of calculation format, for example by making the given presentation format more salient (for tree diagrams equipped with probabilities or natural frequencies in the heroin addiction problem see Figure 1). In sum, rather than systematically varying specific factors (or boosting performance), we wanted (1) to know how participants reason with the materials usually presented in German schools and universities, and (2) to observe a substantial number of people switching or staying with the presentation format in order to analyze their respective reasoning processes. For the same reasons, we implemented standard problem wordings.

Since participants were explicitly asked to write down all calculations they made in order to solve the task, we were able to judge precisely and systematically which format they used for their calculations (see Supplementary Table 2; also see section Coding).

The paper and pencil questionnaire contained a short information paper on the study and some general questions, for example on participants' age or study program, as well as the two tasks. Before participants were allowed to start with the second task, they had to hand in their solution for the first task. Participants were allowed to use a pocket calculator that was provided along with the questionnaire. There was no time limit; on average, participants took approximately 5 min to complete the demographic items and 25 min for both tasks.

### Coding

The normatively correct solutions of the problems were 5% (or 10 out of 200) for the heroin addiction problem and 9.9% (or 55 out of 555) for the car accident problem (the results differ marginally if the task was presented in natural frequencies as opposed to probabilities, e.g., exactly 10% in the car accident probability version vs. 9.9% in the car accident frequency version). In order to guarantee maximum objectivity for classifying the answers as “correct Bayesian inference” or “incorrect Bayesian inference” and also for deciding whether either a probability algorithm or a frequency algorithm had been applied, we used strict coding guidelines (see Supplementary Table 1), which were applied by all coders. Since we were especially interested in whether participants used the correct *algorithm* for solving the task, mere calculation or rounding errors were neglected, resulting in answers that were classified as “correct Bayesian inference” even though the mathematical result was not entirely correct. In the same line, answers that appeared mathematically correct at first glance were classified as “incorrect Bayesian inference” if the result was just incidentally correct, but a wrong algorithm was applied (this rarely happened).

Furthermore, we focused on the cognitive processes underlying each response when determining the “calculation format” of an answer. This cognitive process was measured by analyzing the exact calculations each participant wrote down to come to a solution. When a participant used probabilities (or natural frequencies) only, we classified the solution as “calculated with probabilities” (or natural frequencies, respectively). When both formats were clearly visible in the calculations, we classified the answer according to whether the participant used probabilities or natural frequencies for the *crucial step* in the calculation process, that is, the computation of the denominator in Bayes' formula, as can be seen in equations (1) and (2). Thus, the decisive factor in such unclear cases was the *addition* of two absolute numbers (in favor of a frequency algorithm) or the *multiplication* of probabilities (in favor of a probability algorithm, respectively). If, for example, in the heroin addiction problem a participant used both formats for his or her calculations, but *added* two absolute numbers (e.g., 10 + 190) to obtain the denominator in (2), the answer was classified as “calculated with natural frequencies”. If, on the other hand, a participant used both formats, but *multiplied* two probabilities (e.g., 0.01 × 100%) like in (1) to obtain the respective probabilities for the numerator or the denominator, we classified the answer as “calculated with probabilities” (no participant added frequencies *and* multiplied probabilities).

Two raters coded 21% of all inferences independently according to the coding guidelines (see Supplementary Tables 1, 2). Since in 100% of all cases the correctness was rated in congruence (Cohen's κ = 1; Cohen, 1960), and the calculation format was classified identically in 97% of all cases (Cohen's κ = 0.95), the remaining inferences were rated by one coder.

### Participants

We recruited *N* = 114 students from the University of Regensburg (Bavaria) in summer 2016, and *N* = 69 in winter 2017/2018 (three of which were excluded from the analysis since they had already participated in the study in 2016). Most of these students were enrolled in a teaching math program (*N* = 147), while some of them studied economic information technology, so a certain level of mathematics competency among the participants can be assumed (see also section Discussion). They were at different stages of their studies (most of them in their first two years) and their age ranged from 18 to 38, with an average of 22 years. Out of the total of *N* = 180 participants, 121 were female. Since each participant worked on two tasks, we obtained a total of 360 Bayesian inferences including participants' detailed solution algorithms.

The study was carried out in accordance with the University Research Ethics Standards. Participants were informed that the study was voluntary and anonymous, and no incentives were paid. Participants were asked to give their written informed consent to participate in the study in advance. Thereupon, two students refrained from participating.

## Results

**Figure 2:**
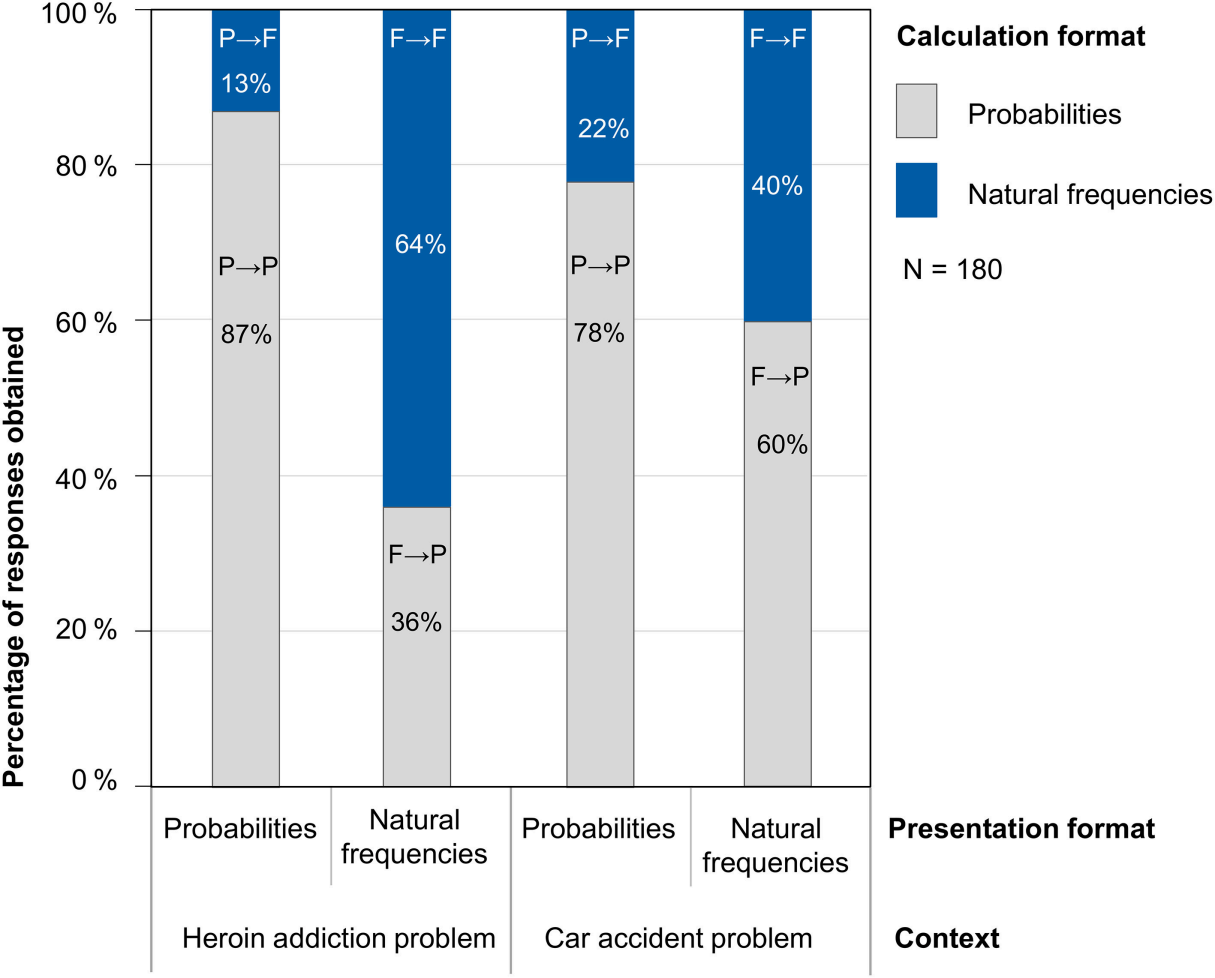
Calculation format by presentation format and context.

In the following, we report the results for the combined sample of *N* = 180 participants, but all detected effects also hold for both the original (*N* = 114) and the replication sample (*N* = 66) independently. As far as our first research question is concerned, the results indeed show a strong preference of participants for calculating with probabilities in both contexts. This is illustrated by Figure 2, where, for example, P→ F denotes participants who were provided with a task in probability format but calculated with natural frequencies. On the one hand, when presented with a task in natural frequency format (second and fourth bars of Figure 2), almost half of participants (49%) nevertheless chose to apply probabilities for their calculations, although the neutral question explicitly allowed for answers in both formats. On the other hand, when they faced a probability version of a task (first and third bars of Figure 2), only 18% across both contexts chose to translate the problem into natural frequencies—despite the explicitly given sample population in the introduction. Taken together, according to our design natural frequencies represented the preferred calculation format in only about one third (34%) of all 360 Bayesian tasks although 50% of all tasks were presented in natural frequency format.

**Figure 3:**
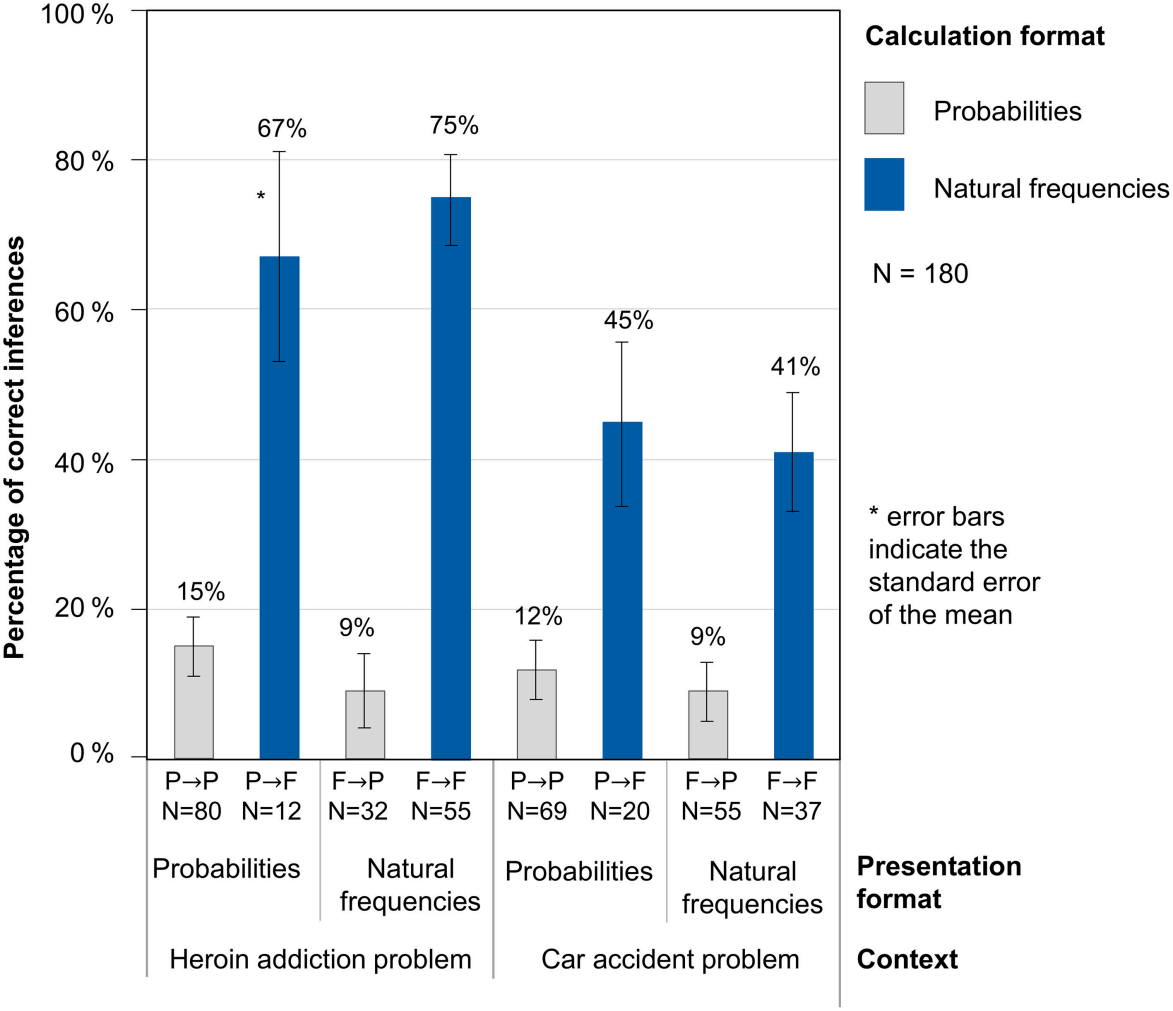
Percentages of correct inferences dependent on the presentation and calculation format in both problems.

While Figure 2 does not yet display performances, Figure 3 shows performance rates in the resulting four combinations of presentation format and calculation format (P→P, P→F, F→F, F→P) for both problem contexts. It becomes clear that when natural frequencies were actively used for the calculations, performance rates were significantly higher than when probabilities were applied. Remarkably, in our design this holds true almost regardless of the presentation format: For both problems, the patterns look very similar for the two presentation formats. The performance in both problems obviously mainly depends on the calculation format, but only to a small amount on the presentation format. In the heroin addiction problem, the difference between both calculation formats is especially pronounced. The highest performance was detected when both variables *presentation format* and *calculation format* were natural frequencies (61% correct responses), descriptively followed by probability tasks that were worked on with frequencies (53% correct responses). In the two other cases (when participants calculated with probabilities), performance rates were considerably lower (13% if the presentation format was probabilities and 9% if the presentation format was natural frequencies).

In general, the beneficial effect of presenting natural frequencies was replicated by our study. While 20% of the Bayesian tasks in probability format were solved correctly across both contexts, the performance rate for the tasks presented in frequency format was 36% (see Table 3). Compared to McDowell and Jacobs (2017), both of these numbers seem rather high. An explanation might lie within our sample: more than 80% of participants were enrolled in a mathematics education program and might therefore have comparably high numeracy, enabling them to perform above average in math tasks (for an analysis of participants' individual differences and switching behavior depending on their cognitive abilities, see below). Note that we also found context effects (36% correct responses in the heroin context vs. 20% correct inferences in the car accident context).

**Table 3:** Percentage of correct Bayesian inferences by context and presentation format (independent of calculation format).

**Presentation format**	**Context**		**Average**
	**Heroin addiction problem**	**Car accident problem**	
Probabilities	22% (*n* = 92 inferences)	19% (*n* = 89 inferences)	20% (*n* = 181 inferences)
Natural frequencies	51% (*n* = 87 inferences)	22% (*n* = 92 inferences)	36% (*n* = 179 inferences)

In order to separate the effects of presentation format and calculation format, we ran a generalized linear mixed model (GLMM) with a logistic link function. Here, we specified probabilities (both as presentation format and as calculation format) as reference category and included the possible explanatory factors “presentation format,” “calculation format” (via dummy coding), and the interaction term of presentation format and calculation format to predict the probability of a correct Bayesian inference in our design.

According to the results of the generalized linear mixed model, the unstandardized regression coefficient for solving a task that was both presented and calculated in probability format was significant (b_0_ = −7.03, SE = 1.32, *z* = −5.32, *p* < 0.001), showing large inter-individual differences (for a discussion of these results, see below). The (unstandardized) regression coefficient for the *presentation format* was non-significant (b_1_ = −3.04, SE = 2.00, *z* = −1.52, *p* = 0.13), whereas the *calculation format* showed a significant regression coefficient (b_2_ = 9.85, SE = 3.85, *z* = 2.56, *p* = 0.01). Finally, the interaction of presentation format and calculation format yielded another significant regression coefficient (b_3_ = 4.85, SE = 2.22, *z* = 2.19, *p* = 0.03), indicating that calculating with natural frequencies increases performance even more when the task is also formulated in natural frequency format (i.e., when the absolute numbers for the frequency algorithm can be directly taken from the problem wording).

The strong differences of individual competencies lead to extreme (unstandardized) regression coefficients in the model. However, a generalized linear model (neglecting inter-individual differences) estimated regression coefficients that—converted into probabilities via the logistic link function—exactly replicated the performance rates found in our data. This is because the GLMM accounts for these large differences in performances by estimating large inter-individual differences between the participants, as the intercepts (denoting the performances when presentation and calculation format was probabilities) were allowed to vary freely between participants. The substantial influence of the inter-individual differences also becomes apparent when inspecting the model fit: Whereas 6.5% of the variance is explained by the fixed GLMM regression coefficients (marginal *R*^2^ = 0.065), the inter-individual differences and the fixed regression coefficients together explain 68.5% of the variance (conditional *R*^2^ = 0.685). However notably, despite the large inter-individual differences, the influence of the fixed effects on the results was clear and strong.

Although we did not explicitly collect data about participants' cognitive abilities (e.g., numeracy, spatial and graphical literacy), these inter-individual differences suggested a closer analysis of our data with this respect. Indeed, we found significant differences in performance especially between two subgroups of our sample: The *N* = 42 mathematics education students aspiring to teach at the academic school track of the German school system (*Gymnasial students*) outperformed the other *N* = 138 participants significantly (50% correct inferences vs. 21%; *t*(358) = 5.294, *p* < 0.001). We assume that this difference is due to the higher numerical, spatial, and graphical abilities of the first group, since they generally outperform the other mathematics education students in mathematics exams or mathematical knowledge tests (e.g., Krauss et al., 2008; see also Lindl and Krauss, 2017, Table 5, p. 396). Moreover, the Gymnasial students receive a considerably more thorough education in mathematics through their study program than the rest of our participants. However interestingly, these differences in cognitive abilities did not have any influence on calculation format preferences. Both subgroups tended in a similar way to prefer using probabilities over natural frequencies for their calculations (32% of Gymnasial students' solutions were based on a frequency algorithm, whereas 35% of the other participants calculated with natural frequencies; *t*(358) = −0.506; *p* = 0.613). As a consequence, although an overall shift of performances might be expected depending on participants' cognitive abilities and education, we assume a certain generalizability of our results across varying abilities and education levels regarding the switching rates (cf. section Discussion).

By examining exploratively participants' reactions on a presented tree diagram, we revealed several instances where the participants had added probabilities to the branches of a tree diagram originally presented with natural frequencies in the nodes. Conversely, only few of the participants equipped a tree diagram that was originally presented in probability format with natural frequencies. When the participants had to construct actively a tree diagram visualizing the textual problem, we detected some instances where already before the diagram was drawn, participants had switched in their calculation format (in both directions: from natural frequencies to probabilities and vice versa). Therefore, some participants translated the presentation format into their calculation format right at the beginning of their problem solution process. However, since we did not systematically test versions without a visualization clue, these findings have to be considered only explorative hints concerning possible cognitive mechanisms that might lead participants to stay with a certain format or to switch from one to the other. These mechanisms will have to be addressed more closely in future research.

## Discussion

In an empirical study with *N* = 180 students from the University of Regensburg, we found that the majority of participants do not actively use natural frequencies in Bayesian reasoning tasks. Even if the task is presented in the intuitive natural frequency format (with a neutral question asking for proportions), about half of the participants still prefer calculating with probabilities instead. Therefore, and since the “standardized” probability format is the “sine qua non” in probability theory, the results of our study reveal the Einstellung effect in Bayesian reasoning situations (Luchins, 1942; Luchins and Luchins, 1959; McCloy et al., 2007). We speculate that such an Einstellung might be enhanced by the still widespread idea that natural frequencies are not “mathematically correct” enough to actually work with in high school and university contexts. As a consequence, participants who might actually notice a possible solution of the Bayesian reasoning task based on a frequency algorithm might still rely on probabilities due to a certain kind of “phobia” to use natural frequencies for their calculations (for a discussion on the impact of affect on overcoming fixed mindsets, see Haager et al., 2014)—despite the ever-growing body of research pointing to the beneficial effects of the frequency concept (e.g., Gigerenzer and Hoffrage, 1995; Barbey and Sloman, 2007; Micallef et al., 2012; Obrecht et al., 2012; Ottley et al., 2016; McDowell and Jacobs, 2017).

Although with our study, we cannot ultimately decide whether the Einstellung effect or this kind of “phobia” lies at the heart of participants' switching back to probabilities, we want to emphasize that both formats are mathematically equivalent in the sense that they can be defined analogously with the same properties and structure. Whatever the case may be, since recent efforts to implement natural frequencies in high school and university curricula appear not to be enough to make people actively take advantage of their benefits, we vouch for an even stronger implementation of the natural frequency concept in secondary education (especially in the higher grades), tertiary education, and in teacher training.

The Einstellung toward preferring probabilities has a negative impact on performance rates: participants working with probabilities perform significantly worse than those who apply natural frequencies for their calculations. Moreover, at least in our design, the calculation format is an even stronger predictor for performance than the presentation format that previous research has mainly concentrated on (e.g., Barbey and Sloman, 2007; Siegrist and Keller, 2011). This suggests that participants who translate natural frequencies into probabilities follow a path that is disadvantageous in two respects: First, they choose the unintuitive probability over the natural frequency format, and second, they are prone to make further mistakes due to translation errors (that we did not explicitly consider in our study). Interestingly, a few participants (18%) did translate probabilities into natural frequencies. This suggests that at least a small minority is to some extent familiar with the natural frequency concept. These participants profit indeed from calculating with natural frequencies since their performance rates increased substantially compared to performances of participants who stay with probabilities (13 vs. 53% across both implemented contexts). This tendency is a first sign that natural frequencies might become an established solution strategy for Bayesian reasoning tasks.

It has to be noted that our sample consisted of university students entirely. Since their mindsets and cognitive abilities (especially numeracy as well as graphical and spatial literacy) probably differ from the general population (Micallef et al., 2012), a different sample might, of course, yield different performance rates. However, we assume that even though the total population might generally perform worse than our sample, those using natural frequencies for their calculations will still outperform those who resort to probabilities. In the same way, we would expect an overall shift of performance rates depending on item difficulty or wording (for factors determining the difficulty of Bayesian reasoning tasks as well as for different problem wordings, see, e.g., Ottley et al., 2016), but we assume relative consistency with respect to format preferences across different Bayesian reasoning tasks. Future research might investigate in detail whether our results indicating an Einstellung effect in Bayesian reasoning situations hold also true when individual differences and item difficulty are systematically controlled.

The context effects in our study in favor of the heroin addiction problem could be explained by having a closer look at the question formulation in the car accident problem. Here, the two relative clauses in the frequency version (see Table 2) demand higher verbal processing abilities and thus make the question harder to understand compared to the frequency question in the heroin addiction problem (only one relative clause, see Table 2). Consequently, the heroin addiction problem presented with natural frequencies yields significantly higher performance rates than the respective version of the car accident problem (51% correct inferences vs. 22%; see Table 3). Moreover, coding in our study was fairly complex (see Supplementary Tables 1, 2), even though we obtained interrater reliability scores of κ = 1 for the correctness of a Bayesian inference and of κ = 0.95 (Cohen, 1960) for determining the calculation format. In addition, we focused only on the correct algorithm applied for classifying an answer as “correct” (see Supplementary Table 1). Thus, we did not concentrate on calculation errors, including those that resulted from translating an information format into the other one. Therefore, we did not systematically detect translation errors dependent on the respective presentation format, in particular. This, however, is a conservative approach, since we assume that more people make mistakes when translating frequencies into probabilities than vice versa.

Furthermore, in an explorative analysis, we detected several instances where the participants had equipped a presented frequency tree diagram with probabilities, suggesting that such a visualization does not prevent the participants from switching from the natural frequency to the probability format for their calculations. We speculate that even the opposite is the case: Since students are familiar with probability tree diagrams but not so much with frequency tree diagrams from their high school careers, the sight of a tree diagram (even though it is equipped with natural frequencies) might trigger their memories of the familiar probability trees and might thus provoke them to fill the diagram with probabilities. Moreover, many participants equipped the tree diagram they had been asked to draw with their chosen calculation format—even if the latter differed from the presentation format. This suggests that the participants tend to decide on their calculation format right at the beginning of their solution process. We thus speculate that the exact moment of the format switch lies immediately after (or even at the same time as) reading the task. Therefore, further research might investigate systematically when exactly people decide on the format they want to use for their calculations and if people possibly alter their decision during the solution process. In addition, it would be interesting to determine whether presenting a visualization such as a tree diagram or actively constructing one enhances or diminishes the Einstellung effect in Bayesian reasoning tasks (e.g., by systematically comparing versions with and without visualization)—and, more generally, whether visualizations affect the calculation format at all.

The question remains open to what extent natural frequencies should be implemented in statistics education, since they can only be used in specific situations (e.g., in Bayesian reasoning problems or tasks where cumulative risk judgment is necessary; see McCloy et al., 2007). We suggest that natural frequencies be taught already at a young age to establish the concept over a longer period of time. When—at a later stage—the focus is shifted more and more to probabilities, a permanent interplay between the two formats seems reasonable. By using natural frequencies to illustrate, for example, the multiplication rule or Bayes' theorem, students can understand the two coexisting formats as equally legitimate representations for the underlying concept of uncertainty. Here, natural frequencies can be used to eliminate typical errors, to make difficult problems more understandable, and to prevent cognitive illusions. When probabilities are presented simultaneously, the connection between the two formats might become more apparent and a deeper understanding of the concept of uncertainty might be achieved. In this respect, future work, for example systematic training studies (cf. Sedlmeier and Gigerenzer, 2001), needs to determine the most successful ways to incorporate natural frequencies in statistics education at secondary and tertiary level in order to overcome the Einstellung effect.

Future research on this topic might also investigate in more detail how much current teachers already know about the frequency concept in order to decide if natural frequencies indeed need a stronger focus in teacher training as we suggest. This could, for example, be realized by systematic teacher interviews. Moreover, future research might address empirically the cognitive mechanisms underlying the Einstellung effect as detected by our study, that is, whether participants assume that a probability algorithm is (a) the only way, (b) the easiest way, or (c) due to a feeling of uneasiness with the frequency concept the only mathematically allowed way to approach the Bayesian problem. Here, qualitative methods such as student interviews might be a valuable tool to clarify situation-specific causes of the Einstellung effect. Finally, it would be interesting to determine effective methods (e.g., visualizations or hints in the problem wording) to prevent people from falling back into probabilities in Bayesian reasoning tasks.

## Data availability statement

The dataset generated can be found on https://epub.uni-regensburg.de/37693/.

## Ethics statement

This study was carried out in accordance with the recommendations of University Research Ethics Standards, University of Regensburg. The protocol was approved by the University of Regensburg. All subjects gave written informed consent in accordance with the Declaration of Helsinki.

## Author contributions

All authors listed have made a substantial, direct and intellectual contribution to the work, and approved it for publication.

### Conflict of interest statement

The authors declare that the research was conducted in the absence of any commercial or financial relationships that could be construed as a potential conflict of interest.
