# Supplementary material for: Why Can Only 24% Solve Bayesian Reasoning Problems in Natural Frequencies: Frequency Phobia in Spite of Probability Blindness
Source: Front Psychol. 2018 Oct 12;9:1833. doi: 10.3389/fpsyg.2018.01833 (PMC6194348; doi:10.3389/fpsyg.2018.01833)
Supplement: Supplementary file 1 [file Table_1.docx]

Supplementary Material

Why can only 24% solve Bayesian reasoning problems in natural frequencies: Frequency phobia in spite of probability blindness

Patrick Weber^1*^, Karin Binder^1^, Stefan Krauss^1^

^1^Mathematics Education, Faculty of Mathematics, University of Regensburg, Regensburg, Germany

*** Correspondence:**

Patrick Weber, Mathematics Education, Faculty of Mathematics, University of Regensburg, Regensburg, Germany.

Patrick.Weber@ur.de

**Supplementary table 1.** Coding guidelines for determining the correctness of a Bayesian inference

| Correctness: | The correctness of a response is classified according to whether the participant used the correct *algorithm* to obtain his or her estimate. |
| --- | --- |
| Correct Bayesian inference: | If the correct algorithm is applied to obtain the estimate, the response is classified as “correct Bayesian inference”.  Factors that determine whether the correct *algorithm* is applied: |
| *If calculation format is probabilities: Bayes’ formula* | - The participant calculated the posterior probability correctly by using Bayes’ formula (10% in the car accident problem and 5% in the heroin addiction problem) - The participant failed to obtain the normatively correct result due to a mistake when translating natural frequencies into probabilities but applied Bayes’ formula correctly - The participant failed to obtain the normatively correct result due to a copying mistake of the numerical information from the task but applied Bayes’ formula correctly - The participant failed to obtain the normatively correct result due to a calculation mistake but applied Bayes’ formula correctly - The participant failed to obtain the normatively correct result due to more than one of the aforementioned calculation or copying mistakes but applied Bayes’ formula correctly |
| *If calculation format is natural frequencies: Addition and division of the correct frequencies* | - The participant calculated the posterior probability correctly by using natural frequencies (55 out of 555 in the car accident problem and 10 out of 200 in the heroin addiction problem)  1. In the car accident problem, the participant applied the frequency algorithm correctly, that is, the participant  - added the two absolute numbers of drivers who cause an accident and are drunk (55) and drivers who do not cause an accident but are still drunk (500) of the sample population to obtain the total of drivers who are drunk (555) - divided the absolute number of drivers who cause an accident and are drunk (55) by the absolute number of drivers who are drunk (555) to obtain the proportion of drivers who cause an accident and are drunk of the drivers who are drunk  1. In the heroin addiction problem, the participant applied the frequency algorithm correctly, that is, the participant  - added the two absolute numbers of heroin addicts with fresh needle pricks (10) and non-addicts of heroin with fresh needle pricks (190) of the sample population to obtain the total of people with fresh needle pricks in this population (200) - divided the absolute number of heroin addicts with fresh needle pricks (10) by the absolute number of people with fresh needle pricks (200) to obtain the proportion of heroin addicts of the people with fresh needle pricks - The participant failed to obtain the normatively correct result due to a mistake when translating natural frequencies into probabilities and/or vice versa but applied the frequency algorithm correctly - The participant failed to obtain the normatively correct result due to a copying mistake of the numerical information from the task but applied the frequency algorithm correctly - The participant failed to obtain the normatively correct result due to a calculation mistake but applied the frequency algorithm correctly - The participant failed to obtain the normatively correct result due to more than one of the aforementioned calculation or copying mistakes but applied the frequency algorithm correctly |
| Incorrect Bayesian inference: | If the correct algorithm is not applied to obtain the estimate, the response is classified as “incorrect Bayesian inference”.  Factors that determine whether the correct *algorithm* is not applied: |
| *If calculation format is probabilities: No application of Bayes’ formula* | The participant did not calculate the posterior probability correctly by using Bayes’ formula (10% in the car accident problem and 5% in the heroin addiction problem) as indicated above |
| *If calculation format is natural frequencies: No addition and division of the correct frequencies* | The participant did not calculate the posterior probability correctly (55 out of 555 in the car accident problem and 10 out of 200 in the heroin addiction problem) by using the frequency algorithm as indicated above |
