# Supplementary material for: Why Can Only 24% Solve Bayesian Reasoning Problems in Natural Frequencies: Frequency Phobia in Spite of Probability Blindness
Source: Front Psychol. 2018 Oct 12;9:1833. doi: 10.3389/fpsyg.2018.01833 (PMC6194348; doi:10.3389/fpsyg.2018.01833)
Supplement: Supplementary file 2 [file Table_2.docx]

Supplementary Material

Why can only 24% solve Bayesian reasoning problems in natural frequencies: Frequency phobia in spite of probability blindness

Patrick Weber^1*^, Karin Binder^1^, Stefan Krauss^1^

^1^Mathematics Education, Faculty of Mathematics, University of Regensburg, Regensburg, Germany

*** Correspondence:**

Patrick Weber, Mathematics Education, Faculty of Mathematics, University of Regensburg, Regensburg, Germany.

Patrick.Weber@ur.de

**Supplementary table 2.** Coding guidelines for determining the calculation format in a Bayesian task

| Calculation format: | The calculation format is classified according to the information format that is predominantly used by a participant to obtain the estimate.  In unclear cases, see the decisive factor at the end of the table |
| --- | --- |
| Probabilities: | If the estimate obtained is calculated predominantly with probabilities, the calculation format is classified as “probabilities”.  Factors that determine whether probabilities are *predominantly* used: |
| *Only probabilities* | The participant did not write down any format other than probabilities |
| *Multiplication of probabilities* | - In the car accident problem, the participant multiplied   - the probability that a driver will cause an accident (1%) and the probability that a driver is drunk, given that he or she causes an accident (55%) to obtain the probability that a driver causes an accident and is drunk (0.55%) and/or   - the probability that a driver will not cause an accident (99%) and the probability that a driver is drunk, given that he or she does not cause an accident (5%) to obtain the probability that a driver does not cause an accident and is drunk (4.95%) and/or   - any other (wrong) pair of probabilities   In mathematical terms:  $P\left( accident and drunk \right)=P\left( accident \right)\cdot P\left( drunk \vert accident \right)=1\%\cdot55\%=0.55\%$  and/or  $P\left( no accident and drunk \right)=P\left( no accident \right)\cdot P\left( drunk \vert no accident \right)=99\%\cdot5\%=4.95\%$   - In the heroin addiction problem, the participant multiplied   - the probability that a person is addicted to heroin (0.01%) and the probability that a person has fresh needle pricks, given that he or she is addicted to heroin (100%) to obtain the probability that a person is addicted to heroin and has fresh needle pricks (0.01%) and/or   - the probability that a person is not addicted to heroin (99.99%) and the probability that a person has fresh needle pricks, given that he or she is not addicted to heroin (0.19%) to obtain the probability that a person is not addicted to heroin and has fresh needle pricks (0.189981%) and/or   - any other (wrong) pair of probabilities   In mathematical terms:  $P\left( heroin addict and needle pricks \right)=P\left( heroin addict \right)\cdot P\left( needle pricks \vert heroin addict \right)=0.01\%\cdot100\%=0.01\%$  and/or  $P\left( no heroin addict and needle pricks \right)=P\left( no heroin addict \right)\cdot P\left( needle pricks \vert no heroin addict \right)=99.99\%\cdot0.19\%=0.189981\%$ |
| *Addition of probabilities* | 1. In the car accident problem, the participant added the probabilities that a driver causes an accident and is drunk (0.55%) to the probability that a driver does not cause an accident and is drunk (4.95%) to obtain the total probability that a driver is drunk (5.5%) and/or added any other (wrong) pair of probabilities   In mathematical terms:  $P\left( drunk \right)=P\left( accident and drunk \right)+P\left( no accident and drunk \right)=0.55\%+4.95\%=5.5\%$   1. In the heroin addiction problem, the participant added the probabilities that a person is addicted to heroin and has fresh needle pricks (0.01%) to the probability that a person is not addicted to heroin and has fresh needle pricks (0.189981%) to obtain the probability that a person has fresh needle pricks (0.199981%) and/or added any other (wrong) pair of probabilities   In mathematical terms:  $P\left( needle pricks \right)=P\left( heropin addict and needle pricks \right)+P\left( no heroin addict and needle pricks \right)=0.01\%+0.189981\%=0.199981\%$ |
| *Division of probabilities* | 1. In the car accident problem, the participant divided the probability that a driver causes an accident and is drunk (0.55%) by the probability that a driver is drunk (5.5%) to obtain the conditional probability that a driver causes an accident, given he or she is drunk (10%) and/or divided any other (wrong) pair of probabilities   In mathematical terms:  $P\left( accident \vert drunk \right)=\frac{P\left( accident and drunk \right)}{P\left( drunk \right)}=\frac{0.55\%}{5.5\%}=10\%$   1. In the heroin addiction problem, the participant divided the probability that a person is addicted to heroin and has fresh needle pricks (0.01%) by the probability that a person has fresh needle pricks (0.199981%) to obtain the conditional probability that a person is addicted to heroin, given he or she has fresh needle pricks (5%) and/or divided any other (wrong) pair of probabilities   In mathematical terms:  $P\left( heroin addict \vert needle pricks \right)=\frac{P\left( heroin addict and neelde pricks \right)}{P\left( needle pricks \right)}=\frac{0.01\%}{0.199981\%}\approx5\%$ |
| *Probability tree* | The participant did not specify his or her calculations but drew a tree diagram equipped with probabilities |
| Natural frequencies: | If the estimate obtained is calculated *predominantly* with natural frequencies, the calculation format is classified as “natural frequencies”.  Factors that determine whether natural frequencies are *predominantly* used: |
| *Only frequencies* | The participant did not write down any format other than frequencies (i.e. absolute numbers) |
| *Addition of frequencies* | 1. In the car accident problem, the participant wrote down probabilities but added the two absolute numbers of drivers who cause an accident and are drunk (55) and drivers who do not cause an accident but are still drunk (500) of the sample population to obtain the total of drivers who are drunk (555) and/or added any other (wrong) pair of frequencies 2. In the heroin addiction problem, the participant wrote down probabilities but added the two absolute numbers of heroin addicts with fresh needle pricks (10) and non-addicts of heroin with fresh needle pricks (190) of the sample population to obtain the total of people with fresh needle pricks in this population (200) and/or added any other (wrong) pair of frequencies |
| *Division of frequencies* | 1. In the car accident problem, the participant divided the absolute number of drivers who cause an accident and are drunk (55) by the absolute number of drivers who are drunk (555) to obtain the proportion of drivers who cause an accident and are drunk of the drivers who are drunk (regardless of whether the participant then gave the estimate as a proportion, in decimal numbers, percent, or in frequency format) and/or divided any other (wrong) pair of frequencies 2. In the heroin addiction problem, the participant divided the absolute number of heroin addicts with fresh needle pricks (10) by the absolute number of people with fresh needle pricks (200) to obtain the proportion of heroin addicts of the people with fresh needle pricks (regardless of whether the participant then gave the estimate as a proportion, in decimal numbers, percent, or in frequency format) and/or divided any other (wrong) pair of frequencies |
| *Frequency tree* | The participant did not specify his or her calculations but drew a tree diagram equipped with absolute numbers |
| **Decisive factor:** | If at least one factor indicating probabilities and one factor indicating natural frequencies as calculation format are evident in a participant’s solution, the determining factor for probabilities is whether he or she multiplied probabilities, whereas the determining factor for frequencies is whether he or she added frequencies. There was no participant who multiplied probabilities but added natural frequencies as described above |
| General remark: | If a participant wrote down only an answer (without specifying his or her calculations) or did not write down anything at all, the calculation format was classified the same as the presentation format |
